# Impact on ART initiation of point-of-care CD4 testing at HIV diagnosis among HIV-positive youth in Khayelitsha, South Africa

**DOI:** 10.7448/IAS.16.1.18518

**Published:** 2013-07-04

**Authors:** Gabriela EM Patten, Lynne Wilkinson, Karien Conradie, Petros Isaakidis, Anthony D Harries, Mary E Edginton, Virginia De Azevedo, Gilles van Cutsem

**Affiliations:** 1 MSF Khayelitsha, Cape Town, South Africa; 2 MSF Mumbai, India; 3 London School of Hygiene and Tropical Medicine, London, UK; 4 International Union Against Tuberculosis and Lung Disease, Paris, France; 5 City of Cape Town, Health Directorate, Khayelitsha, Cape Town, South Africa; 6 MSF Cape Town, South Africa; 7 University of Cape Town, Cape Town, South Africa

**Keywords:** HIV/AIDS, youth and adolescents, antiretroviral therapy, attrition, South Africa, pre-antiretroviral therapy care, point-of-care CD4 testing, operational research

## Abstract

**Introduction:**

Despite the rapid expansion of antiretroviral therapy (ART) programmes in developing countries, pre-treatment losses from care remain a challenge to improving access to treatment. Youth and adolescents have been identified as a particularly vulnerable group, at greater risk of loss from both pre-ART and ART care. Point-of-care (POC) CD4 testing has shown promising results in improving linkage to ART care. In Khayelitsha township, South Africa, POC CD4 testing was implemented at a clinic designated for youth aged 12–25 years. We assessed whether there was an associated reduction in attrition between HIV testing, assessment for eligibility and ART initiation.

**Methods:**

A before-and-after observational study was conducted using routinely collected data. These were collected on patients from May 2010 to April 2011 (Group A) when baseline CD4 count testing was performed in a laboratory and results were returned to the clinic within two weeks. Same-day POC CD4 testing was implemented in June 2011, and data were collected on patients from August 2011 to July 2012 (Group B).

**Results:**

A total of 272 and 304 youth tested HIV-positive in Group A and Group B, respectively. Group B patients were twice as likely to have their ART eligibility assessed compared to Group A patients: 275 (90%) vs. 183 (67%) [relative risk (RR)=2.4, 95% CI: 1.8–3.4, *p*<0.0001]. More patients in World Health Organization (WHO) Stage 1 disease (85% vs. 69%), with CD4 counts≥350 cells/µL (58% vs. 35%) and more males (13% vs. 7%) were detected in Group B. The proportion of eligible patients who initiated ART was 50% and 44% (*p*=0.6) in Groups B and A, respectively; and 50% and 43% (*p*=0.5) when restricted to patients with baseline CD4 count≤250 cells/µL. Time between HIV-testing and ART initiation was reduced from 36 to 28 days (*p*=0.6).

**Discussion:**

POC CD4 testing significantly improved assessment for ART eligibility. The improvement in the proportion initiating ART and the reduction in time to initiation was not significant due to sample size limitations.

**Conclusions:**

POC CD4 testing reduced attrition between HIV-testing and assessment of ART eligibility. Strategies to improve uptake of ART are needed, possibly by improving patient support for HIV-positive youth immediately after diagnosis.

## Introduction

The rapid expansion of antiretroviral therapy (ART) programmes in the last decade has greatly improved access to HIV treatment; however, this expansion has been hampered with high rates of attrition. Of particular concern are the poor retention rates observed between HIV diagnosis and treatment initiation [1], resulting in patients accessing ART at a later, more advanced stage of disease. Much of this attrition occurs between HIV diagnosis and assessment for ART eligibility [2,3], indicating that many HIV-positive patients leave care without the knowledge of their need for treatment. Recently, it has been shown that the use of point-of-care (POC) CD4 cell tests for immunological assessment may reduce loss to follow-up before initiation of treatment [4].

The HIV care needs of youth and adolescents are among the most challenging to address. With an estimated 5 million young people in 2009 aged 15–24 years living with HIV globally [5], this vulnerable group has been identified as being at particularly high risk of poor adherence to treatment [6,7], loss to follow-up and treatment failure [8], worsening their long-term prognosis. HIV-positive youth have also been identified as being at greater risk of attrition prior to ART initiation compared with adults [9]. There is now growing recognition of the need to focus efforts towards retaining this group in HIV care. However, there is little documented worldwide experience concerning youth-oriented approaches to HIV prevention, diagnosis, care and treatment.

In Khayelitsha township, Cape Town, South Africa, separate clinics for young people aged between 12 and 25 years (youth clinics) have been introduced and offer youth-friendly services to address the needs of this difficult population group [1–8]. In June 2011, POC CD4 cell-count testing was introduced in a youth clinic, to address the problem of high levels of loss to follow-up between HIV diagnosis, assessment for ART eligibility and ART initiation.

The aim of this study was to determine whether POC CD4 testing in HIV-positive youth in Khayelitsha, South Africa, was associated with reduced attrition from HIV testing to ART initiation.

## Methods

### Study design

This was a before-and-after observational cohort study, using routinely collected data.

### Study setting

The study was undertaken in Khayelitsha, a peri-urban township on the outskirts of Cape Town, South Africa. Khayelitsha, with a population of about 500,000 inhabitants, has one of the highest burdens of HIV in the country, and in 2011 the antenatal HIV prevalence was measured at 37% [10].

Since 2005, MSF has been providing support to the City of Cape Town (CoCT) health services at two youth clinics in Khayelitsha. The study site, Site C Youth Clinic, currently offers the following services: HIV testing and counselling, enrolment on ART, diagnosis and treatment of sexually transmitted infections, family planning services, termination of pregnancy services and general care. According to clinic records, the clinic currently tests approximately 340 youth per month with an HIV prevalence of 8%. At the end of October 2012, 484 patients were in HIV care, 293 of whom were on ART. The majority of patients in youth clinics are horizontally infected, while adolescents who were infected through mother-to-child transmission continue to be followed at the general ART clinics.

### ART management

In South Africa, ART eligibility is determined by World Health Organization (WHO) clinical staging combined with CD4 cell count. Patients with WHO Stage 4 disease or those with a CD4 count below a specified threshold are eligible for ART. In the Western Cape, the CD4 count threshold level of 250 cells/µL was in use until the end of August 2011, when the South African guidelines were changed and a new threshold level of 350 cells/µL was set [11,12].

**Figure 1:**
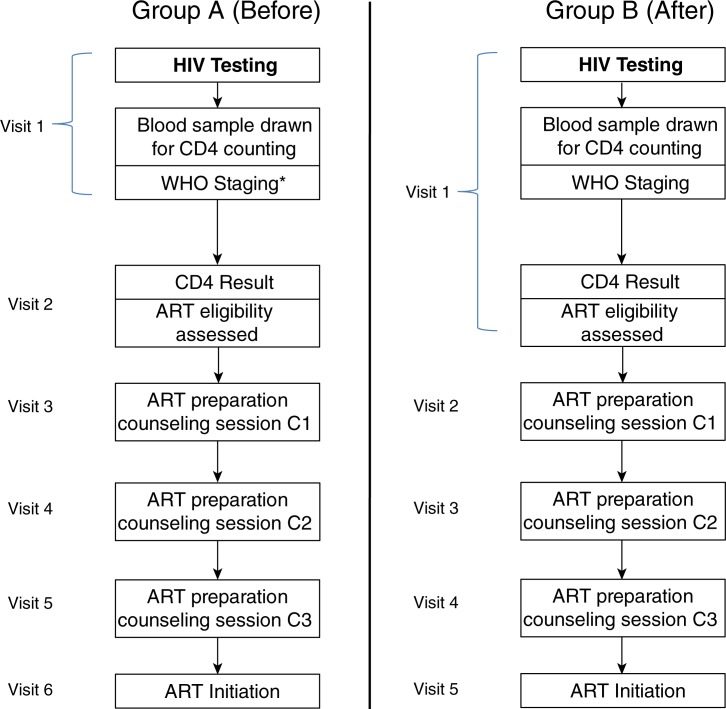
Steps between HIV testing and ART initiation before and after the introduction of POC CD4 testing at a youth clinic in Khayelitsha, South Africa 2010–2012. *Those with WHO Stage 4 begin ART preparation counselling at their next clinic visit, and do not wait for the CD4 count.

Once a patient has tested HIV-positive, there are several steps leading to assessment for ART eligibility. On the day of HIV testing, bloods are drawn to ascertain baseline CD4 count, and WHO staging is performed. Prior to June 2011, baseline CD4 count testing was performed at the public-sector laboratory in central Cape Town, situated at around 30 km from the clinic. Blood samples were taken at the clinic and sent to the laboratory for investigation. CD4 count testing was performed using a Beckman Coulter XL flow cytometer (Beckman Coulter, Brea, CA, USA). Paper-based results generally took up to two weeks to return to the clinic. In June 2011, a POC CD4 machine (Alere Pima CD4, Waltham, MA, USA) was installed in the clinic and is only used to assess baseline CD4 count. Patients testing positive for HIV have their CD4 cell count test done on the same day. ART eligibility assessment is established within 20 min, as compared with the two-week wait requiring a second visit to the clinic when laboratory-based testing was used. Patients receive post-CD4 cell count counselling while waiting for their results. Eligible patients undergo three ART preparation counselling sessions. The scheduling of these sessions is at weekly intervals following eligibility. Patients completing the counselling sessions may initiate ART. The steps between HIV testing and ART initiation, as well as the changes in scheduling to this process that occurred after the introduction of POC CD4 counting are summarized in Figure 1. All patients receive CD4 count testing, however those who have WHO Stage 4 disease, and are therefore eligible for ART regardless of their CD4 count, do not have to wait for the CD4 result to proceed to ART preparation counselling.

### Study participants

Retrospective data on patients testing HIV-positive prior to the installation of the POC machine were collected from 1 May 2010 to 30 April 2011. A period of three months for implementation of the model was allowed. Data of patients testing HIV-positive after the intervention were introduced were collected from 1 August 2011 to 31 July 2012.

Study periods were chosen based on sample size estimates. We estimated that for a two-sided significance level of 95% and a ratio of exposed to unexposed of 1:1, a sample size of 62 in each group was needed to achieve 80% power in detecting a 25% difference in the proportion initiating ART before and after the intervention.

### Data collection and analysis

Data were collected between June and September 2012 and variables included: age, sex, baseline CD4 count, WHO stage, attendance at pre-ART counselling sessions and short-term retention in clinical care following ART initiation. Data were collected from paper HIV-testing registers and from patient folders into a structured proforma using Epidata [13]. Study outcomes were compared between the two groups (Group A – when baseline CD4 testing was done using laboratory methods prior to POC CD4 testing and Group B – when baseline POC CD4 testing was used). These outcomes included: i) receiving a CD4 cell count test result, ii) assessment of ART eligibility, iii) attending each of the ART preparation counselling sessions, iv) eligible patients initiating ART and v) alive and retained in ART care at two months on treatment. Loss to follow-up between HIV testing and ART initiation was defined as having no recorded subsequent visit within three months of the patients recorded last visit. The Chi-square test, risk ratios (RR) and 95% confidence intervals (CI) were used to compare outcomes between the two groups. The time from HIV diagnosis to CD4 count and to ART initiation was compared by calculating the median number of days between HIV-testing and ART initiation. Wilcoxon rank sum test was used to compare medians. Data were analyzed using STATA (StataCorp, College Station, TX, USA) statistical software version 10. Levels of significance were set at 5%.

### Ethics approval

Ethics approval was obtained from the University of Cape Town Human Research Ethics Committee. This study has met the Ethics Advisory Group of the International Union against Tuberculosis and Lung Disease and the MSF Ethics Review Board-approved criteria for analysis of routinely-collected programme data. Permission to do the study was obtained from the CoCT's Research committee.

## Results

A total of 3605 and 3973 individuals were tested for HIV in the two study periods, respectively. In Group A (before), 272 (7.5%) youth patients tested HIV-positive and in Group B (after), 304 (7.7%) tested positive. Baseline characteristics of HIV-positive patients in Groups A and B are summarized in Table 1.

**Table 1:** Comparison of baseline characteristics in patients testing HIV-positive, before (Group A) and after (Group B) point-of-care CD4 testing was introduced at a youth clinic in Khayelitsha, South Africa, 2010–2012

	Group A	Group B	*p*
Patients tested HIV-positive: *n*	272	304	
Median age in years at HIV test (Interquartile Range)	22.4 (20.5–24.0)	22.6 (20.6–23.9)	0.8
Gender: *n* (%)			0.0386
Female	237 (87)	246 (81)	
Male	19 (7)	41 (13)	
Unknown	16 (6)	17 (6)	
Reason for HCT: *n* (%)			0.0755
Voluntary testing	105 (39)	99 (33)	
STI treatment	67 (25)	88 (29)	
Termination of pregnancy	48 (18)	68 (22)	
Family planning	49 (18)	44 (14)	
Other	0 (0)	4 (1)	
Unknown	3 (1)	1 (0)	
WHO clinical stage: *n* (%)			0.0002
Stage 1	189 (69)	257 (85)	
Stage 2	20 (7)	12 (4)	
Stage 3	5 (2)	7 (2)	
Stage 4	3 (1)	1 (0)	
Unknown	55 (20)	27 (9)	
CD4 count: *n* (%)			<0.0001
<200	28 (10)	20 (7)	
200–249	18 (7)	15 (5)	
250–349	42 (15)	63 (21)	
>350	94 (35)	177 (58)	
Not recorded	90 (33)	29 (10)	
Median baseline CD4 count in cells/µL (interquartile range)	355 (242–491)	414 (316–548)	

HCT=HIV counselling and testing; STI=sexually transmitted infections; WHO=World Health Organization.

Patients testing HIV-positive in Group B had a higher frequency of WHO Stage 1 disease and CD4 counts above 350 cells/µL, and were more likely to be males than patients in Group A.

**Figure 2:**
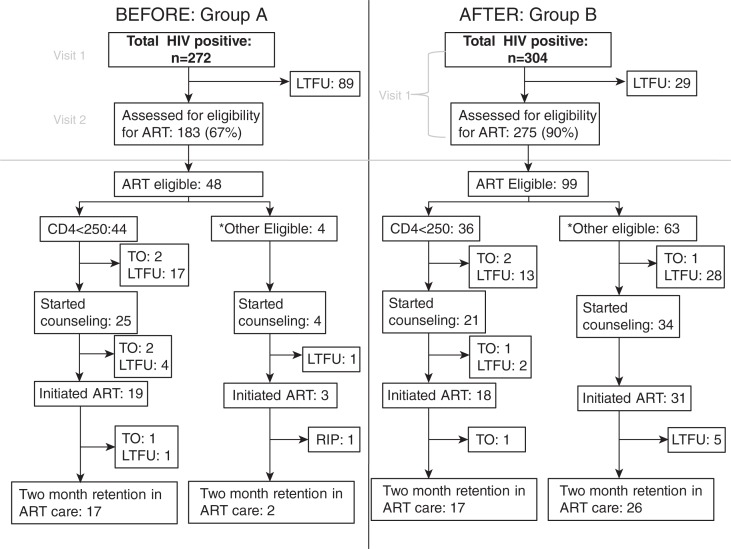
Attrition from HIV testing to antiretroviral therapy (ART) initiation before (Group A) and after (Group B) introduction of point-of-care CD4 testing. at a youth clinic in Khayelitsha, South Africa 2010–2012. TO=transfer out; LTFU=loss to follow-up. *Patients with CD4≥250 or WHO Stage 4.

More patients in Group B received clinical staging (RR=1.70, 95% CI: 1.24–2.34, *p*<0.001). Other outcomes of the study are summarized in Figure 2.

Patients with WHO Stage 4 disease were eligible for ART regardless of their CD4 count and were excluded from the rest of the analysis. In Group B, more patients received their CD4 cell count test results and had their ART eligibility assessed (90% vs. 67%, RR=2.4, 95% CI: 1.8–3.4, *p*<0.0001). In Group A, 44 of the 183 (24%) who were assessed for ART eligibility had a CD4 count below 250, compared with 36 of the 275 (13%) in Group B. For those with a recorded CD4 count, the median number of days from HIV test to receiving a CD4 count result was 14 days (range 2–404 days) in Group A, compared with 0 days (range 0–14) in Group B.

Among those eligible for ART with a CD4 count<250 cell/µL, there was neither significant difference in the proportion starting ART preparation counselling sessions (56% vs. 58%, *p*=0.9) nor in completing all three counselling sessions (50% vs. 56%, *p*=0.5) in Group A vs. Group B, respectively.

The proportion of eligible patients who initiated ART was 44% and 50% (*p*=0.6) in Group A and Group B, respectively and a similar proportion were retained on therapy at three months after initiation (RR=1.0, 95% CI: 0.8–1.2, *p*=0.9). When restricting the comparison to patients with a baseline CD4≤250 cells/µL, 43% and 50% initiated ART in Groups A and B, respectively (*p*=0.5). There was no difference in the proportion of ART-eligible males initiating ART compared to females (47% vs. 50%, *p*=0.8).

In Group A, 38% of eligible patients initiated ART within 90 days of HIV-testing, compared with 40% in Group B (*p*=0.6). For those who initiated ART within 90 days of HIV testing, the median number of days between HIV testing and ART initiation was 36 and 28 in Groups A and B, respectively (*p*=0.6).

## Discussion

This is one of the first assessments of pre-treatment losses to care amongst HIV-positive youth, and the first assessment of POC CD4 testing on the uptake of ART in this population group. In summary, significantly more patients received their CD4 count test result and had their ART eligibility assessed following the implementation of POC CD4 testing. Loss to follow-up was high in both groups, with half of the eligible patients in each of the groups lost from care prior to ART initiation, the majority prior to starting ART preparation counselling. There was an improvement in the proportion initiating ART after the introduction of POC CD4 testing; however, this was not significant. In both groups, a similar proportion of eligible patients started and completed ART preparation counselling sessions. There was an eight-day reduction in the time from HIV testing to ART initiation in Group B, but this reduction was not statistically significant. POC CD4 testing did result in a two-week reduction in time from HIV-testing to ART eligibility assessment.

Two previous studies, in Mozambique and South Africa, have shown that the use of POC CD4 testing in primary care facilities reduced the overall attrition prior to ART initiation and reduced time to ART initiation [2,4]. Similar to our findings, the study in Mozambique showed no difference in the proportion of patients lost to follow-up between ART eligibility and ART initiation. In particular, results from the Mozambique study for those aged 15–29 years showed that 54% of patients in total were lost from care between clinic enrolment up to ART initiation, findings similar to those obtained in our study. The study in South Africa also found those aged 19–25 years were more likely to be lost from care prior to initiation of ART compared with older age groups. Two systematic reviews on retention in care from HIV-testing to ART initiation in sub-Saharan Africa found that of those eligible for ART, only two-thirds initiated ART [1,14]. These reviews included studies which considered all eligible patients or an adult-only population. By comparison, our findings suggest that youth experience even higher attrition prior to ART initiation.

While there was an increase in ART uptake following the introduction of POC CD4 testing, this was not statistically significant. This study shows that for HIV-positive youth there is high attrition prior to initiating ART, warranting further research into simplifying and reducing the lengthy ART initiation process. Therefore, further qualitative research to explore the reasons for such high attrition among youth prior to commencing ART is needed.

At the time when laboratory CD4 testing methods were used, the two-week period between HIV testing and receiving CD4 count results left newly diagnosed HIV-positive patients who failed to return for follow-up care with an uncertain prognosis. Without the knowledge of their need to start treatment, patients who would have qualified for ART, might have preferred to ignore their HIV diagnosis and delay returning to the clinic, whilst continuing in a state of denial about their health. A POC CD4 test ensures that a healthcare provider can immediately inform a patient of the importance of starting treatment thereby optimizing the opportunity to empower the patient with both knowledge of their HIV status and the need to start treatment. Awareness of CD4 count and eligibility for treatment may lead to uptake of ART services before becoming sick or severely immune suppressed.

Our findings indicate that HIV-positive youth may be most at risk of attrition immediately after HIV diagnosis, since in both study groups high rates of attrition occurred after the clinic visit at which the patient was first diagnosed. Patients may prefer to test for HIV away from their usual place of residence for reasons of confidentiality. Other patient characteristics and programme factors found to be associated with attrition prior to ART initiation include stigma and fear of disclosure [9]. In both study groups, most patients who returned to the clinic following their HIV diagnosis went on to complete the ART preparation counselling sessions and initiated ART. ART preparation counselling could be adjusted so that the first counselling session occurs on the same day as HIV diagnosis and eligibility assessment.

Whilst youth appear to be at greater risk, compared to adults, of attrition prior to ART initiation, POC CD4 testing is likely to benefit this vulnerable group in the same way it benefits adults, by shortening and simplifying the process from HIV testing to ART initiation. Patient factors specific to youth, rather than health service delivery factors, may therefore need to be addressed, if pre-ART attrition is to be reduced for this group.

The strengths of this study are that it focused on a population group known for its problems with retention in care and the study was conducted within the arena of routine patient care. The results should therefore be representative of the situation on the ground. The design of the study also allows insights into attrition prior to ART initiation in this group, which is a subject of national and international importance.

There were several limitations. First, this study was limited by a small sample size. The loss of one of the clinic's registers meant that the desired sample size was not achieved. This limited our ability to interpret the results and draw conclusions from the observed small improvement in the proportion of ART-eligible youth initiating ART and the reduction in days between HIV-testing and ART initiation. Second, the changes to the national guidelines during the study period, increasing the CD4 threshold for ART eligibility from 250 to 350 cells/µL, make it impossible to make direct comparisons between the two groups of the effect of POC CD4 testing on the number and proportion of patients who were ART eligible after HIV testing. Third, temporal changes to the baseline characteristics of those testing HIV-positive at the clinic has resulted in patients in Group B having higher CD4 counts compared with those in Group A, reducing the sample which can be directly compared with Group A patients. Fourth, in the period after the package of care was introduced at the clinic, facility-based nurses were trained and mentored to manage and initiate ART patients. Prior to this, only a doctor was initiating patients on ART. This change may have altered ART initiation processes, and could be another reason for the improvements in clinical staging that we observed in the second group of patients. Finally, even after the installation of POC CD4 testing, some patients did not receive their CD4 results on the day of their HIV test, possibly due to the machine not functioning due to lack of cartridges. The results of this study may not be generalisable to all youth, as in most settings youth access ART care from primary care clinics for all ages, and thus their overall attrition rates may differ [1,2,4,9,14].

## Conclusion

POC CD4 testing improved ART eligibility assessment, optimized the opportunity to provide a prognosis to the patient at HIV diagnosis, and reduced attrition between HIV-testing and ART eligibility. The small sample size of this study limited our ability to ascertain if the observed improvements in uptake of ART and time to initiation following the introduction of POC testing were real. Further research and strategies are needed to retain youth patients immediately after HIV diagnosis and ensure that these patients are retained in care and initiate ART when required.
